# Eplerenone, diabetes, and chronic kidney disease in patients hospitalized for acute heart failure: findings from the EARLIER trial

**DOI:** 10.1186/s12933-025-02659-y

**Published:** 2025-03-22

**Authors:** Masatake Kobayashi, Akira Yamashina, Kazuhiro Satomi, Masataka Watanabe, Ryu Takagi, Ayako Tezuka, Shin Ito, Masanori Asakura, Masafumi Kitakaze

**Affiliations:** 1 Department of Cardiology, Tokyo Medical University, 6-7-1, Nishi-shinjuku, Shinjuku, Tokyo, Japan; 2 Miyabi Heart and Care Clinic, Tokyo, Japan; 3 Department of Clinical Research and Development, National Cerebral and Cardiovascular Center, Osaka, Japan; 4 Department of Cardiovascular and Renal Medicine, Hyogo Medical University, Nishinomiya, Hyogo Japan; 5 Hanwa Memorial Hospital, Osaka, Japan

**Keywords:** Acute heart failure, Eplerenone, Mineralocorticoid receptor antagonist, Diabetes, Chronic kidney disease, Prognosis

## Abstract

**Background:**

Mineralocorticoid receptor antagonists (MRAs) are often underutilized in patients with heart failure (HF), particularly those with diabetes and/or chronic kidney disease (CKD). However, the impact of concurrent diabetes and CKD on the efficacy and safety of eplerenone in acute HF remains uncertain.

**Methods:**

The EARLIER trial enrolled patients with acute HF, who were randomized to receive eplerenone or placebo for 6 months. Patients were categorized based on the presence of diabetes and/or CKD (defined by eGFR < 45 ml/min/1.73 m^2^ or UACR ≥ 30 mg/g), and the associations between diabetes/CKD categories and cardiovascular outcomes were assessed. The effects of eplerenone on HF-related outcomes (i.e., cardiovascular death, HF hospitalization, worsening HF, or out-of-hospital diuretic intensification) and adverse events were also assessed across diabetes/CKD status.

**Results:**

Among 300 patients (mean age 67 ± 13 years; 73% male), 39% had diabetes, mean estimated glomerular filtration rate was 63 ± 18 ml/min/1.73 m^2^, median urine albumin-to-creatinine ratio was 34 mg/g (13–84 mg/g), and 58% had CKD. Patients with both diabetes and CKD (26%) had a higher risk of cardiovascular death and/or hospitalization compared to those without either disease (HR, 95% CI = 2.57, 1.29–5.12; *P* = 0.007, *P*-for-interaction = 0.049), and poor prognosis persisted after adjusting for covariates (i.e., natriuretic peptide) (adjusted-HR, 95% CI = 2.33, 1.12–4.84; *P* = 0.02). Furthermore, the effects of eplerenone on HF-related outcomes and adverse events were consistent regardless of diabetes/CKD categories (all-*P*-for interaction > 0.05).

**Conclusions:**

In patients with acute HF, the combination of diabetes and CKD was associated with an increased risk of cardiovascular events. However, the efficacy and safety of eplerenone were not influenced by diabetes and CKD status.

**Graphical abstract:**

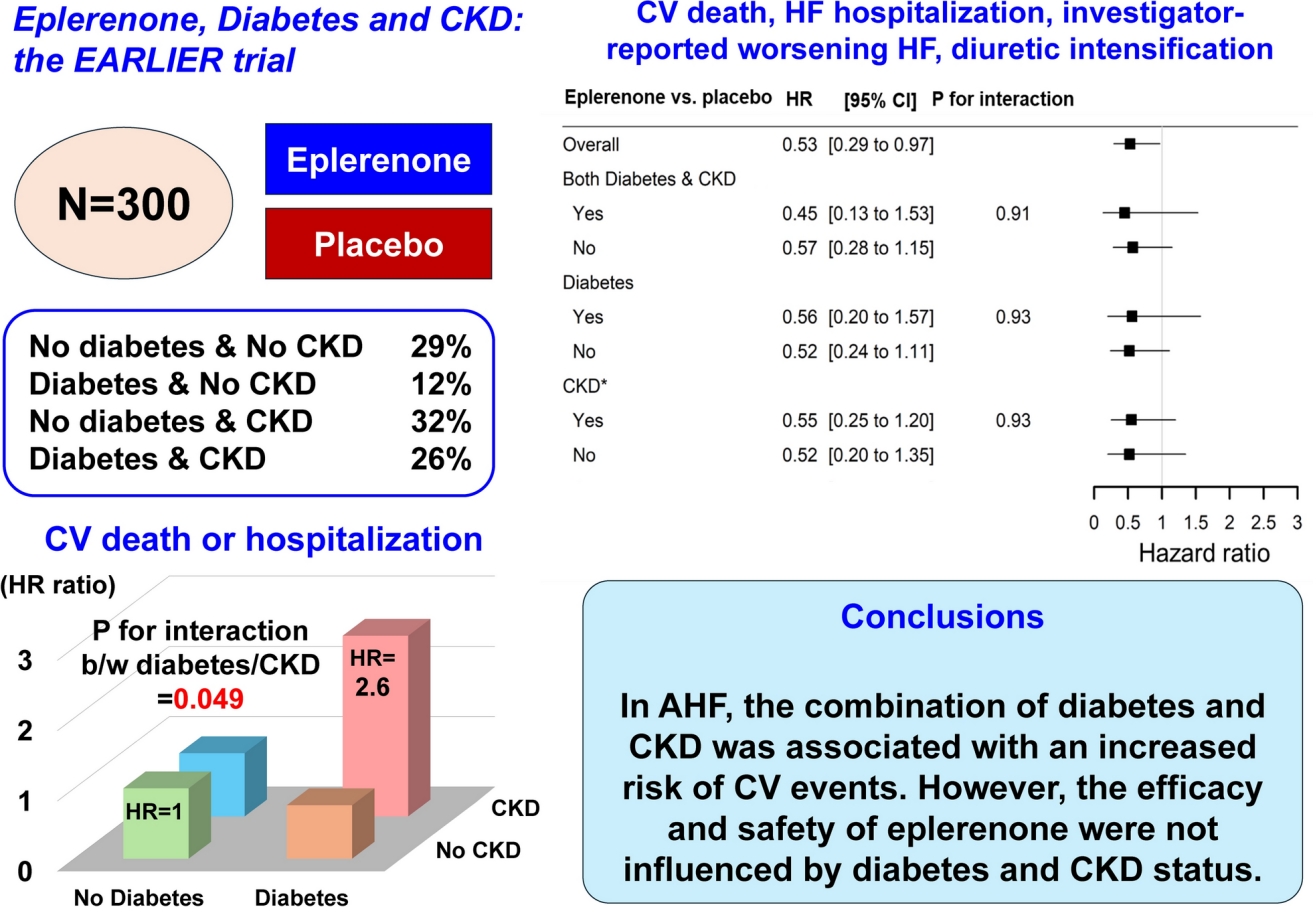

**Supplementary Information:**

The online version contains supplementary material available at 10.1186/s12933-025-02659-y.

## Introduction

Diabetes and chronic kidney disease (CKD) are common comorbidities in patients with heart failure (HF) and a reduced ejection fraction (HFrEF). Together, these conditions synergistically increase the risk of cardiovascular (CV) morbidity and mortality [1–3]. For patients hospitalized with acute HF, the presence of these comorbidities may influence disease severity, treatment response, and overall prognosis.

Eplerenone, a mineralocorticoid receptor antagonist (MRA), is a cornerstone of evidence-based therapy for improving patient outcomes in HFrEF [4, 5] or left ventricular systolic dysfunction after myocardial infarction [6]. However, registry data showed that MRA therapy was often underutilized in HFrEF [7, 8], particularly for patients with both diabetes and CKD. The under-prescription is likely due to the perceived concerns about MRA-associated adverse events, particularly the risk of hyperkalemia [8–10]. The concern may be amplified in patients hospitalized for acute HF who often require frequent medication adjustments and dose changes [11, 12]. Furthermore, it remains unclear whether the effects of eplerenone on HF-related outcomes and renal outcome differ across diabetes/CKD status.

The aims of the present analysis were to describe the characterization and prognosis of diabetes and CKD in patients with acute HF, and to assess whether the efficacy and safety of eplerenone are influenced by baseline presence of diabetes and CKD.

## Methods

### Study population

The EARLIER trial was a multicenter, randomized, double-blind, placebo-controlled study (JMACCT clinical trials registry identifier: JMA-IIA00127), which included 300 patients hospitalized for acute HF in Japan. Participants were randomized to receive either eplerenone or placebo at any point from 3 to 14 days after HF hospitalization, with the aim of evaluating the impact of eplerenone on clinical outcomes over a 6-month period. The rationale, design and main results have been published previously [13, 14]. Acute HF was defined as the presence of at least one following signs: pulmonary rales, radiographic pulmonary congestion and a third heart sound. Key exclusion criteria were a serum potassium concentration > 5.0 mmol/L, or an estimated glomerular filtration rate (eGFR, Chronic Kidney Disease Epidemiology Collaboration [15]) < 30 mL/min/1.73 m^2^. Study drug was initiated at 25 mg/day and increased to 50 mg/day, while, for eGFR 30–50 mL/min/1.73 m^2^, it was initiated at 25 mg every other day and increased to 25 mg/day, maintaining serum potassium < 5.0 mmol/L. If down-titrated or discontinued due to eGFR or serum potassium, it was resumed once potassium dropped below 5.0 mmol/L.

For the present analysis, patients were categorized as having diabetes if they were on antidiabetic drugs 3 months before the enrollment or if baseline HbA1c ≥ 6.5%, while CKD were considered if either baseline eGFR < 45 mL/min/1.73 m^2^ or baseline urine albumin creatinine ratio (UCAR) > 30 mg/g.

All patients provided written informed consent before enrollment in the study. This study was conducted according to the Declaration of Helsinki and, the Institutional Review Board at each institution approved this trial.

### Statistical analysis

Categorical variables are described as frequencies (percentages) and continuous variables are described as means ± standard deviation or median (25th and 75th percentiles) depending on the variable distributions. Comparisons of characteristics across diabetes and CKD categories were analyzed using analysis of variance, Kruskal–Wallis and χ^2^ tests, as appropriate.

Two Cox regression models were used to assess the impact of diabetes and CKD on CV events (i.e., CV death and CV hospitalization), and to evaluate the effect of eplerenone on HF-related events across diabetes/CKD categories. As previously published, for HF-related events, we used a composite outcome of CV death, HF re-hospitalization, worsening HF (based on investigator reports in the case report form), and out-of-hospital diuretic intensification (any increase in the furosemide-equivalent diuretic dose during the post-discharge period) [16, 17]. An interaction test was performed to assess whether baseline diabetes and CKD status would influence the response to eplerenone.

For renal outcomes, we tested whether eplerenone affected UACR levels during follow-up, using analysis of covariance (ANCOVA) to compare the difference in changes between eplerenone and placebo groups, separately in patients with both diabetes/CKD and those without both diseases.

For eplerenone-related adverse events, we assessed the rates of worsening renal function (WRF, > 20% or 30% decrease of eGFR), hyperkalemia (potassium > 5.5 or 6.0 mmol/L), and hypotension (systolic blood pressure < 90 mmHg) throughout the follow-up period [17]. Additionally, based on the study protocol, investigators recorded adverse events specifically associated with eplerenone (i.e., WRF, hyperkalemia, hypotension, and volume depletion/ dehydration) [17]. Interaction between eplerenone and diabetes/CKD categories on the adverse events was also tested.

Statistical analyses were performed using R version 4.2.2 (R Development Core Team, Vienna, Austria). Statistical significance was defined as a two-sided *p*-value < 0.05.

## Results

### Patient characteristics

In a total of 300 patients included, mean age was 67 ± 13 years, 73% were males, mean body mass index was 25 ± 5 kg/m^2^, 39% had diabetes, 60% had New York Heart Association (NYHA) III or IV, mean eGFR was 63 ± 18 ml/min/1.73 m^2^ and median UACR was 34 mg/g (25th and 75th centiles; 13 to 84). Within 72 h of hospitalization for acute HF, 69.4% of patients were initiated on eplerenone, and median doses of the study drug throughout the trial were 37.5 mg (25.0 to 42.5) in the eplerenone group and 40.0 mg (25.0 to 45.0) in the placebo group.

Among these patients, 29%, 12%, 32% and 26% of patients had neither diabetes nor CKD, diabetes without CKD, CKD without diabetes and both diabetes and CKD, respectively. Patients with both diabetes and CKD had more frequently CV diseases, higher blood pressure, higher UACR levels and walked shorter compared to those with either disease alone or those without either disease (all-*P* values < 0.05) Table 1*.*

**Table 1:** Patient characteristics across diabetes and CKD categories

	No Diabetes & No CKD (N = 87)	Diabetes & No CKD (N = 37)	No Diabetes & CKD (N = 96)	Diabetes & CKD (N = 79)	*p*-value
Age, years	65.8 ± 10.8	63.6 ± 11.8	68.5 ± 15.4	67.3 ± 12.5	0.16
Men, N (%)	64 (73.6%)	29 (78.4%)	63 (65.6%)	61 (77.2%)	0.28
Body mass index, kg/m^2^	23.6 ± 4.0	25.5 ± 6.1	24.2 ± 5.6	25.9 ± 5.9	0.059
NYHA ≥ III, N (%)	49 (56.3%)	16 (43.2%)	64 (66.7%)	51 (64.6%)	0.063
Medical history, N (%)					
Hypertension	57 (65.5%)	25 (67.6%)	75 (78.1%)	71 (89.9%)	**0.002**
Myocardial infarction	10 (11.5%)	10 (27.0%)	10 (10.4%)	27 (34.2%)	**< 0.001**
Atrial fibrillation	38 (43.7%)	11 (29.7%)	36 (37.5%)	20 (25.3%)	0.077
Stroke	10 (11.5%)	6 (16.2%)	5 (5.2%)	18 (22.8%)	**0.006**
Prior history of HF	5 (5.7%)	3 (8.1%)	7 (7.3%)	17 (21.5%)	**0.004**
Medications, N (%)					
ACEi or ARB	65 (74.7%)	31 (83.8%)	69 (71.9%)	67 (84.8%)	0.14
Beta-blocker	62 (71.3%)	25 (67.6%)	56 (58.3%)	48 (60.8%)	0.27
Diuretic	79 (90.8%)	30 (81.1%)	87 (90.6%)	75 (94.9%)	0.13
Thiazide/thiazide-like	6 (6.9%)	5 (13.5%)	8 (8.3%)	15 (19.0%)	0.062
Anti-diabetic drug	0 (0.0%)	19 (51.4%)	0 (0.0%)	37 (46.8%)	**< 0.001**
Insulin	0 (0.0%)	3 (8.1%)	0 (0.0%)	10 (12.7%)	**< 0.001**
Statin	32 (36.8%)	19 (51.4%)	31 (32.3%)	50 (63.3%)	**< 0.001**
Aspirin	32 (36.8%)	21 (56.8%)	28 (29.2%)	40 (50.6%)	**0.005**
Systolic BP, mmHg	114.0 ± 19.5	116.2 ± 21.3	122.3 ± 18.2	125.8 ± 21.7	**< 0.001**
Heart rate, bpm	82.3 ± 20.2	85.2 ± 13.5	88.5 ± 21.4	86.3 ± 16.4	0.087
Laboratory data					
Hemoglobin, g/dL	13.7 ± 1.9	13.4 ± 2.0	13.7 ± 2.0	13.3 ± 2.0	0.40
Sodium, mmol/L	140.3 ± 2.8	139.3 ± 2.6	140.6 ± 2.6	140.0 ± 2.7	0.077
Potassium, mmol/L	4.0 ± 0.4	4.0 ± 0.4	3.9 ± 0.4	3.9 ± 0.4	0.58
eGFR, ml/min/1.73m^2^	65.4 ± 13.0	75.3 ± 17.4	59.3 ± 17.4	59.9 ± 21.1	**< 0.001**
BNP, pg/mL	310 (121–539)	341 (190–585)	485 (308–711)	380 (184–702)	**0.006**
UACR, mg/g	11.6 (5.6–17.5)	13.3 (6.9–19.5)	66.0 (40.0–131.8)	98.8 (44.4–316.4)	**< 0.001**
Echocardiogram					
LVEF, %	29.4 ± 7.4	30.9 ± 8.2	29.8 ± 6.8	31.3 ± 8.2	0.38
LVSD, mm	49.4 ± 9.2	49.1 ± 9.3	49.2 ± 8.7	48.9 ± 7.8	0.98
E/e' ratio	16.2 ± 6.9	20.4 ± 21.5	18.2 ± 11.3	18.3 ± 9.1	0.64
TRPG, mmHg	31.3 ± 11.8	28.8 ± 10.5	35.5 ± 12.2	33.1 ± 16.0	0.06
6MWT, m	448.2 ± 119.1	393.2 ± 118.0	377.7 ± 130.0	383.0 ± 129.9	**0.001**
KCCQ OSS	55.4 (30.5–75.0)	63.0 (38.0–76.8)	55.3 (35.3–79.9)	56.8 (36.5–79.7)	0.87
Eplerenone allocation, N (%)	39 (44.8%)	18 (48.6%)	49 (51.0%)	42 (53.2%)	0.73

Values are expressed as mean ± SD, n (%) or median (25th to 75th percentile)

*CKD* chronic kidney disease, *NYHA* New York Heart Association, *HF* heart failure, *ACEi* angiotensin converting enzyme inhibitor, *ARB* angiotensin receptor blocker, *BP* blood pressure, *eGFR* estimated glomerular filtration rate, *BNP* B-type natriuretic peptide, *UACR* urine albumin creatinine ratio, *LVEF* left ventricular ejection fraction, *LVDS* left ventricular end-systolic diameter, *TRPG* tricuspid regurgitation pressure gradient, *6MWT* six-minute wall test, *KCCQ-OSS* Kansas city cardiomyopathy questionnaire overall summary score

Bold values indicate significant difference

### Associations of diabetes/CKD categories with clinical outcomes

Over a 6-month period, 18.1% of patients experienced CV death and/or hospitalization. Considering patients without diabetes or CKD as a reference group, those with either disease alone did not have a significantly increased risk of CV death and/or hospitalization (both *p*-values > 0.10) (Table 2). However, those with both diabetes and CKD had a significantly higher risk of CV death and/or hospitalization (HR [95% CI] = 2.57 [1.29 to 5.12], *P* < 0.01; *P*-for-interaction = 0.049), and the poor prognosis persisted even after adjusting for covariates (i.e., natriuretic peptide) (adjusted-HR [95% CI] = 2.33 [1.12 to 4.84]; *P* = 0.02) (Table 2).

**Table 2:** Association of diabetes/CKD categories with cardiovascular death and/or hospitalization

	Model 1		Model 2		Model 3		*P*-for-interaction between diabetes and CKD
	HR (95% CI)	*P*-value	HR (95% CI)	*P*-value	HR (95% CI)	*P*-value	
No Diabetes & No CKD	(reference)		(reference)		(reference)		**0.049**
Diabetes & No CKD	0.76 (0.24 to 2.35)	0.63	0.73 (0.24 to 2.28)	0.59	0.67 (0.21 to 2.12)	0.49	
No Diabetes & CKD	0.90 (0.40 to 1.99)	0.79	0.91 (0.41 to 2.04)	0.82	0.92 (0.41 to 2.09)	0.84	
Diabetes & CKD	2.57 (1.29 to 5.12)	**0.007**	2.50 (1.25 to 4.98)	**0.10**	2.33 (1.12 to 4.84)	**0.02**	

HR, hazard ratio; CI, confidence interval

Model 1, unadjusted model

Model 2, adjusted for age and sex

Model 3, adjusted for age, sex, prevalence of hypertension and atrial fibrillation, prior heart failure hospitalization, estimated glomerular filtration rate and b-type natriuretic peptide at baseline

Bold values indicate significant difference

### Effect of eplerenone on HF outcome or UACR across diabetes/CKD categories

**Fig. 1:**
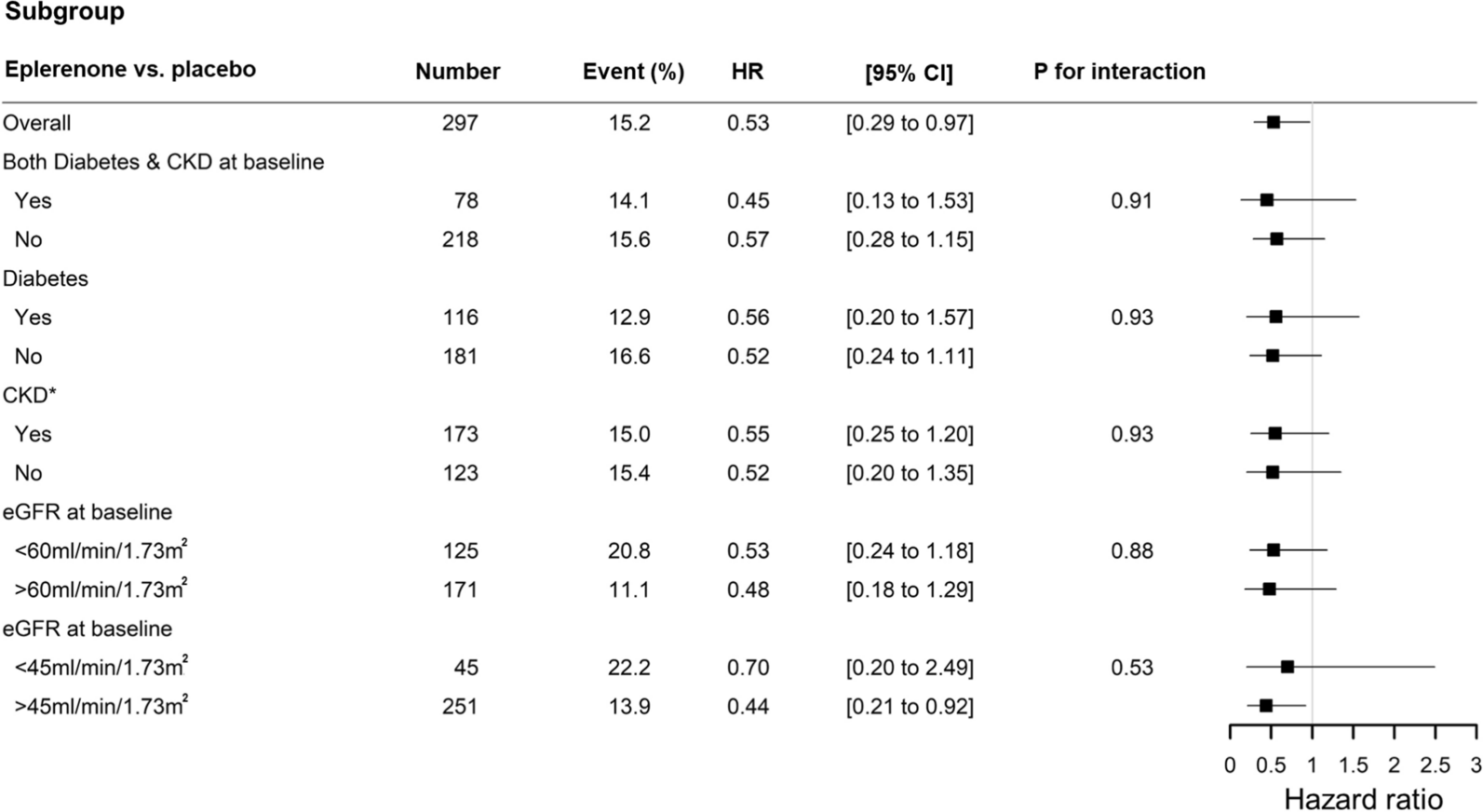
Subgroup analysis of the effect of eplerenone versus placebo on HF-related outcomes across diabetes and kidney disease categories. *CKD included eGFR at baseline < 45 ml/min/1.73 m^2^ or UACR at baseline > 30 mg/g. HR, hazard ratio; CI, confidence interval; CKD, chronic kidney disease; eGFR, estimated glomerular filtration rate; UACR, urine albumin creatinine ratio

Eplerenone versus placebo significantly reduced the risk of the composite of CV death, HF hospitalization, worsening HF or out-of-hospital diuretic intensification over a 6-month period (HR [95%CI] = 0.53 [0.29 to 0.97]; *P* = 0.04) (Fig. 1). The benefit from eplerenone initiation was consistent irrespective of the status of diabetes and/or CKD (all-*P*-for-interaction > 0.10) (Fig. 1).

Furthermore, the effects of eplerenone on such HF-related events were consistent regardless of whether initiation occurred within or after 72 h of hospitalization for acute HF (*P*-for-interaction > 0.10).

Focusing on the effect of eplerenone on UACR levels, patients with both diabetes and CKD had higher UACR levels than those without both diseases (98.8 mg/g [44.4 to 316.4] versus 19.8 mg/g [8.7 to 54.9]). Over the 6-month period, a significant interaction was observed between eplerenone and the presence of both diabetes and CKD in relation to changes in UACR levels from baseline to week 12 (*P-*for-interaction = 0.02). Among patients with both diabetes and CKD, eplerenone resulted in a greater numerical reduction in UACR levels than those without both diseases (− 252.8 mg/g [− 572.2–66.6] versus − 9.7 mg/g [− 33.7 to 4.3]) (Supplemental Table 1).

### Safety of eplerenone across subgroups with diabetes and kidney disease

Overall, eplerenone versus placebo did not increase the rates of MRA-associated adverse events (i.e., WRF, hypotension, and volume depletion/ dehydration) except for mild hyperkalemia (serum potassium > 5.5 mmol/L) (7.4% in eplerenone group versus 1.3% in placebo group; *P* = 0.01). However, the rate of severe potassium (serum potassium > 6.0 mmol/L) was similar between the treatment groups (*P* = 0.47) (Table 3). Importantly, risks of these eplerenone-associated adverse events were not modified by any criteria of diabetes and CKD (all-*P*-for-interaction > 0.10) (Table 3). Furthermore, patients who were on thiazide/thiazide-like diuretics at baseline did not have an increased risk of eplerenone-associated adverse events (all *P* for interaction > 0.10).

**Table 3:** Eplerenone-associated adverse events across presence of diabetes/kidney disease

	Placebo (N = 151)	Eplerenone (N = 149)	*P*-value	*P*-for-interaction with diabetes/CKD categories	*P*-for-interaction with diabetes	*P*-for-interaction with CKD	*P*-for-interaction with GFR < 60
Worsening renal function							
eGFR drop > 20%	61 (40.4%)	60 (40.3%)	0.98	0.63	0.80	0.54	0.87
eGFR drop > 30%	25 (16.6%)	26 (17.4%)	0.84	0.32	0.88	0.30	0.58
Investigator-reported WRF	17 (11.3%)	25 (16.8%)	0.17	0.15	0.30	0.23	0.63
Hyperkalemia							
Potassium > 5.5 mmol/L	2 (1.3%)	11 (7.4%)	0.01	0.99	0.99	0.99	0.56
Potassium > 6.0 mmol/L	0 (0.0%)	2 (1.3%)	0.47	0.99	0.99	0.99	0.99
Investigator-reported hyperkalemia	8 (5.3%)	13 (8.7%)	0.24	0.15	0.16	0.30	0.09
Hypotension							
Systolic BP < 90 mmHg	33 (21.9%)	35 (23.5%)	0.74	0.43	0.39	0.18	0.16
Investigator-reported low BP	10 (6.6%)	12 (8.1%)	0.63	0.43	0.31	0.16	0.47
Volume depletion or dehydration							
Investigator-reported volume depletion or dehydration	27 (17.9%)	23 (15.4%)	0.57	0.34	0.58	0.14	0.76

Abbreviations are presented in Table 1

## Discussion

In Japanese patients hospitalized for acute HF, we have shown that those with both diabetes and CKD had more frequent CV diseases, limited exercise tolerance, and had a higher risk of CV events compared to patients with either diabetes or CKD. However, eplerenone significantly reduced risk of HF-related events regardless of diabetes or CKD status and numerically lowered UACR levels, particularly in patients with both diabetes and CKD. Additionally, the risk of adverse events was not modified by diabetes or CKD status. These findings suggest that comorbid diabetes and CKD increased the risk of CV events; however, the efficacy and safety of eplerenone remain consistent across diabetes/CKD categories, encouraging clinicians to initiate eplerenone treatment in patients hospitalized for acute HF.

In the present analysis, the prevalence of diabetes, CKD, and their combination was comparable to that observed in patients with acute HF in previously published data [18–20]. Although several prior analyses showed a high risk of CV events in patients with either diabetes or CKD, prognosis of patients with acute HF who had combined diabetes and CKD remains unclear. In the large sized registry from the US, which included > 350,000 patients with acute HF, patients with lower admission eGFR levels had higher risk of in-hospital mortality as well as had more often a prevalence of diabetes [21]. In the real-world data in the United Kingdom, which included > 90,000 patients identified with HF by electronic health records, the risk of CV events was the highest in combined diabetes and CKD, compared with either disease alone [22]. Diabetes and CKD may synergistically increase intraglomerular pressure, damage glomerular membranes, and promote inflammation and fibrosis in CV tissues [23, 24]. The mechanism may be particularly accelerated in patients with acute HF, who likely experience hemodynamic imbalance and hormonal activation [11, 25]. Importantly, in the present analysis, even after adjustment for natriuretic peptides, as a well-established prognostic marker, the combined diabetes and CKD was associated with worse outcomes in patients with acute HF, underscoring the importance of closely monitoring patients with these comorbidities.

Eplerenone may be underutilized for the treatment of acute HF particularly in patients with both diabetes and CKD, likely due to safety concerns rather than a lack of efficacy. In the Eplerenone Post-Acute Myocardial Infarction Heart Failure Efficacy and Survival Study (EPHESUS), which included 6,642 patients with left ventricular ejection fraction ≤ 40% after myocardial infarction, eplerenone, compared to placebo, reduced the risk of the primary cardiovascular outcome. The benefits of eplerenone treatment were consistent regardless of diabetes or CKD status [6]. However, in the subsequent analysis of the EPHESUS trial, a prevalence of diabetes and eGFR ≤ 60 ml/min/1.73 m^2^ were independent predictors of having hyperkalemia, defined as serum potassium ≥ 6.0 mmol/L [26]. The comorbidities were also associated with the risk of hyperkalemia in patients with relatively stable HFrEF who were treated with eplerenone, from the Eplerenone in Mild Patients Hospitalization and Survival Study in Heart Failure (EMPHASIS-HF) [5, 27]. Similarly, in patients with severe HFrEF on spironolactone, from the Randomized Aldactone Evaluation Study (RALES) trial, diabetes and CKD increased risk of hyperkalemia (serum potassium ≥ 5.5 mmol/L) in multivariable model [28]. Results of other prior studies also suggested that diabetes and CKD increased risk of hyperkalemia in patients hospitalized for HF [29, 30]. Reflecting the perceived safety concerns, registry data showed diabetes and/or CKD were associated with a lower likelihood of MRA prescription [31]. However, the results presented here show that the risk of hyperkalemia was not significantly modified by diabetes and CKD status in acute HF, suggesting a lower-than-expected risk of hyperkalemia associated with eplerenone in patients with diabetes and CKD.

We observe that eplerenone numerically reduced UACR levels over 12 weeks, particularly in patients with both diabetes and CKD. In a meta-analysis of 14 studies involving 1,193 adults with eGFR > 15 ml/min/1.73 m^2^ and proteinuria, spironolactone or eplerenone reduced the magnitude of proteinuria compared with placebo or standard care over a median duration of 3.5 months [32]. Similarly, among patients with diabetes and UACR ≥ 50 mg/g who were on enalapril, eplerenone significantly reduced UACR levels over 12 weeks: 41.0% with eplerenone 50 mg/day, 48.4% with eplerenone 100 mg/day, compared to 7.4% with placebo [33]. Importantly, given that the degree of congestion substantially impacts UACR levels, our findings suggest that the reno-protective effects of eplerenone may persist for 12 weeks, even after eplerenone initiation during hospitalization for acute HF [34].

The guidelines recommend the use of finerenone, non-steroidal MRA, for the treatment of diabetes and CKD [35, 36], which was evidenced from the robust efficacy of finerenone in the Finerenone in Reducing Kidney Failure and Disease Progression in Diabetic Kidney Disease (FIDELIO-DKD) and the Finerenone in Reducing Cardiovascular Mortality and Morbidity in Diabetic Kidney Disease (FIGARO-DKD) trials [37, 38]. For safety concerns, finerenone was expected to have a lower risk of hyperkalemia than steroidal MRAs based on different pharmacological data (i.e., tissue distribution) [39] and results of head-to-head comparison in a prior trial [40]. However, in this trial, finerenone was treated at relative low dose (≤ 20 mg/day), and when finerenone was used up to 40 mg/day in a population of HF and preserved ejection fraction, hyperkalemia risk was similar to what was reported in other steroidal MRAs [41, 42]. Additionally, in a meta-analysis, which included 1,838 patients with diabetes and CKD from 17 randomized trials, eplerenone showed numerically similar risk of hyperkalemia compared to finerenone, and its lower risk than spironolactone [43]. Although serum potassium should be routinely monitored in patients treated with eplerenone, our findings support its initiation in patients with acute HF and comorbid diabetes and CKD, to ensure they are not deprived of the benefits of disease-modifying therapies [1]. Further clinical trials exploring the efficacy of eplerenone in patients with this high-risk population are warranted.

### Limitations

The results presented should be interpreted within the context of several potential limitations. This study is a post-hoc analysis of randomized controlled trial with a moderate sample size, relatively low event rates, and a prolonged hospital stay (2–3 weeks) conducted in Japan [44]. We lacked available information on diabetes types (e.g., type 1 or type 2), which may affect our findings [45]. Furthermore, during the trial period, sodium-glucose co-transporter-2 (SGLT2) inhibitors, in addition to sacubitril-valsartan, had not yet been approved. SGLT2 inhibitors are now a cornerstone of guideline-recommended therapy for improving outcomes and potentially mitigating hyperkalemia risk in patients with HF, CKD, and diabetes [1, 36, 46–48]. Therefore, the lack of SGLT2 inhibitors in our study may impact our results, highlighting the need for further investigation with contemporary data.

## Conclusions

In patients hospitalized for acute HF, one-fourth had both diabetes and CKD, which significantly increased the risk of cardiovascular events compared to those without either disease. However, the efficacy and safety of eplerenone were not affected by diabetes or CKD status. These findings may encourage clinicians to initiate eplerenone treatment in patients with acute HF who have these non-cardiovascular comorbidities.

## Supplementary Information

Supplementary Material 1

## Data Availability

No datasets were generated or analysed during the current study.
