# Supplementary material for: Eplerenone, diabetes, and chronic kidney disease in patients hospitalized for acute heart failure: findings from the EARLIER trial
Source: Cardiovasc Diabetol. 2025 Mar 22;24:136. doi: 10.1186/s12933-025-02659-y (PMC11929252; doi:10.1186/s12933-025-02659-y)
Supplement: Supplementary file 1 — Supplementary Material 1 [file 12933_2025_2659_MOESM1_ESM.docx]

**Supplemental materials**

**Supplemental table 1. Changes in Urine Albumin-to-creatinine Ratio by Eplerenone Across Presence of Diabetes/CKD**

|  | No Both Diabetes and CKD | |  | Both Diabetes and CKD | | P-for-interaction between treatment effect and Diabetes/CKD |
| --- | --- | --- | --- | --- | --- | --- |
|  | **Placebo** | **Eplerenone** |  | **Placebo** | **Eplerenone** |  |
|  | **Median (95% CI)** | **Median (95% CI)** |  | **Median (95% CI)** | **Median (95% CI)** |  |
| Baseline |  |  |  |  |  |  |
| UACR, mg/g | 19.8 (8.6 – 52.0) | 20.1 (8.8 – 65.8) |  | 71.6 (54.1 – 298.6) | 108.3 (54.1 – 316.4) |  |
| Week 4 |  |  |  |  |  |  |
| UACR, mg/g | 9.4 (5.6 – 20.0) | 10.4 (5.5 – 18.3) |  | 42.5 (14.2 – 85.1) | 27.2 (9.5 – 117.9) |  |
| Mean difference (95% CI)  from baseline to week 4 | -10.9 (-31.0 to 9.2) | |  | -70.7 (-168.2 to 26.9) | | 0.16 |
| P-value | P=0.29 | |  | P=0.16 | |  |
| Week 7 |  |  |  |  |  |  |
| UACR, mg/g | 11.2 (5.6 – 30.2) | 12.3 (6.4 – 30.1) |  | 37.8 (12.8 – 102.9) | 25.4 (9.0 – 214.7) |  |
| Mean difference (95% CI)  from baseline to week 7 | -43.7 (-112.5 to 25.1) | |  | -153 (-400.2 to 92.6) | | 0.17 |
| P-value | P=0.21 | |  | P=0.23 | |  |
| Week 12 |  |  |  |  |  |  |
| UACR, mg/g | 11.1 (5.1 – 32.4) | 9.4 (5.1 – 25.6) |  | 51.3 (21.9 – 94.2) | 24.8 (7.2 – 141.3) |  |
| Mean difference (95% CI)  from baseline to week 12 | -9.7 (-33.7 to 4.3) | |  | -252.8 (-572.2 to 66.6) | | **0.02** |
| P-value | P=0.43 | |  | P=0.13 | |  |

CKD, chronic kidney disease; UACR, urine albumin creatinine ratio; CI, confidence interval.
